# *Bifidobacterium animalis* subsp. *lactis* V9 improves quality of life in advanced gastrointestinal cancer through gut microbiota–metabolite modulation

**DOI:** 10.1093/ismeco/ycag127

**Published:** 2026-05-13

**Authors:** Liuqing Yang, Yanfang Liu, Jingjin Li, Jiahua Lv, Qi Zhang, Minghua Cong, Hanping Shi, Heping Zhang

**Affiliations:** Inner Mongolia Key Laboratory of Dairy Biotechnology and Engineering, Inner Mongolia Agricultural University, Hohhot 010018, Inner Mongolia, China; Key Laboratory of Dairy Products Processing, Ministry of Agriculture and Rural Affairs, Inner Mongolia Agricultural University, Hohhot 010018, Inner Mongolia, China; Key Laboratory of Dairy Biotechnology and Engineering, Ministry of Education, Inner Mongolia Agricultural University, Hohhot 010018, Inner Mongolia, China; Department of Clinical Nutrition, Beijing Shijitan Hospital, Capital Medical University, Beijing 100038, China; Inner Mongolia Key Laboratory of Dairy Biotechnology and Engineering, Inner Mongolia Agricultural University, Hohhot 010018, Inner Mongolia, China; Key Laboratory of Dairy Products Processing, Ministry of Agriculture and Rural Affairs, Inner Mongolia Agricultural University, Hohhot 010018, Inner Mongolia, China; Key Laboratory of Dairy Biotechnology and Engineering, Ministry of Education, Inner Mongolia Agricultural University, Hohhot 010018, Inner Mongolia, China; Inner Mongolia Key Laboratory of Dairy Biotechnology and Engineering, Inner Mongolia Agricultural University, Hohhot 010018, Inner Mongolia, China; Key Laboratory of Dairy Products Processing, Ministry of Agriculture and Rural Affairs, Inner Mongolia Agricultural University, Hohhot 010018, Inner Mongolia, China; Key Laboratory of Dairy Biotechnology and Engineering, Ministry of Education, Inner Mongolia Agricultural University, Hohhot 010018, Inner Mongolia, China; Department of Radiotherapy, Sichuan Cancer Hospital, Chengdu, Sichuan 610041, China; Department of Colorectal Surgery, Zhejiang Cancer Hospital, Hangzhou 310022, China; Department of Comprehensive Oncology, National Cancer Center/National Clinical Research Center for Cancer/Cancer Hospital Chinese Academy of Medical Sciences and Peking Union Medical College, Beijing 100021, China; Department of Clinical Nutrition, Beijing Shijitan Hospital, Capital Medical University, Beijing 100038, China; Center for Clinical Nutrition and Department of Colorectal Surgery, The First Affiliated Hospital of Wenzhou Medical University, Wenzhou, Zhejiang 325000, China; Key Laboratory of Cancer FSMP for State Market Regulation, Beijing 100820, China; Inner Mongolia Key Laboratory of Dairy Biotechnology and Engineering, Inner Mongolia Agricultural University, Hohhot 010018, Inner Mongolia, China; Key Laboratory of Dairy Products Processing, Ministry of Agriculture and Rural Affairs, Inner Mongolia Agricultural University, Hohhot 010018, Inner Mongolia, China; Key Laboratory of Dairy Biotechnology and Engineering, Ministry of Education, Inner Mongolia Agricultural University, Hohhot 010018, Inner Mongolia, China

**Keywords:** bile acids, chemotherapy toxicity, esophageal cancer, gastric cancer, gut–brain axis, metabolomics, probiotic intervention

## Abstract

Chemotherapy for advanced gastric and esophageal cancer is often limited by severe gastrointestinal and systemic toxicities that profoundly impair patients’ quality of life. We conducted a randomized, double-blind, placebo-controlled trial in 104 patients to evaluate whether *Bifidobacterium animalis* subsp. *lactis* V9 (V9; 2 × 10^10^ CFU/day) mitigates these effects. Participants received V9 or placebo daily for 18 weeks alongside standard chemotherapy. Supplementation with V9 significantly improved EORTC QLQ-C30 scores for overall health status, fatigue, nausea, vomiting, appetite loss, cognitive functioning, role functioning, and insomnia (all *P* < .01). Integrated metagenomic and metabolomic analyses of stool samples revealed that V9 did not alter overall microbial α- or β-diversity but induced targeted shifts: it enriched beneficial taxa, such as *B. pseudocatenulatum*, *Agathobacter rectalis*, and *Lachnospira hominis*, while depleting pathobionts such as *Fusobacterium varium* and *Enterocloster clostridioformis*. These microbial changes correlated with favorable metabolic reprogramming, including increased fecal levels of pyridoxamine, 5′-methylthioadenosine, and palmitoylcarnitine, as well as decreased levels of taurine-conjugated bile acids and several amino acids (*P* < .05). Critically, these metabolite alterations were significantly associated with clinical improvements. Our findings demonstrate that V9 enhances quality of life during chemotherapy not through global microbiota restructuring, but via precise modulation of functionally relevant bacteria and their metabolic outputs. This supports V9 as a mechanistically grounded, targeted adjuvant therapy to improve resilience and well-being in patients with advanced upper gastrointestinal cancers.

## Introduction

Gastrointestinal cancers represent a leading cause of global cancer incidence and mortality, imposing a substantial public health burden. Among them, gastric and esophageal cancer are particularly prevalent malignancies of the digestive tract, characterized by persistently high rates of morbidity and death [1, 2]. Owing to their nonspecific early symptoms, most patients are diagnosed only at advanced stages, which severely limits therapeutic options and compromises clinical outcomes. Beyond the direct effects of the disease, patients with advanced gastrointestinal tumors frequently endure a constellation of treatment-related adverse effects. Chemotherapy and radiotherapy, cornerstones of systemic management, often induce significant gastrointestinal distress, including nausea, vomiting, diarrhea, abdominal distension, constipation, and loss of appetite [3–5]. Compounded by symptoms such as pain, fatigue, and immunosuppression, these side effects accelerate nutritional decline and weight loss, ultimately diminishing treatment tolerance, worsening quality of life, and potentially contributing to treatment discontinuation and poor prognosis [6–8].

Emerging evidence underscores the gut microbiota as a central mediator of host physiology and therapeutic response in cancer [9–11]. Dysbiosis, microbial imbalance, has been implicated in the pathogenesis and progression of gastrointestinal malignancies [12, 13]. Moreover, cytotoxic chemotherapy not only targets tumor cells but also disrupts the gut mucosa, impairing barrier function and triggering systemic inflammation [14, 15]. This iatrogenic dysbiosis may enrich pro-inflammatory or pathogenic taxa while depleting beneficial commensals, thereby exacerbating gastrointestinal symptoms and undermining patient well-being during treatment. Consequently, strategies aimed at preserving or restoring gut microbial homeostasis has gained increasing attention as potential means to mitigate chemotherapy toxicity and improve clinical outcomes. It is noteworthy that treatment responses to advanced gastric and esophageal cancers are influenced by a variety of factors, including not only microbiome-related factors but also tumor molecular characteristics [16], host immune function [17], and nutritional status [18]. Therefore, while modulating gut microbiota homeostasis is a promising adjuvant therapy strategy, it should be considered within the broader context of host–tumor–immune interactions, as these factors collectively determine treatment outcomes.

Probiotics, defined live microorganisms that confer health benefits when administered in adequate amounts, represent a promising avenue for modulating the gut ecosystem during cancer therapy [19]. Clinical studies demonstrate that probiotics can enhance intestinal barrier function, promote mucosal repair, suppress pathobionts, and enrich the beneficial ones [20, 21]. Unlike symptomatic treatments targeting isolated gastrointestinal complaints, probiotics act through system-wide modulation of the microbiota and its functional output. Accumulating data indicate that probiotic supplementation can alleviate chemotherapy-induced diarrhea and constipation [22–24]; ameliorate cancer-related cognitive impairment [25, 26]; and improve mood, sleep, and postoperative recovery, likely through the gut–brain axis [27, 28]. Despite these advances, most existing studies remain confined to phenotypic observations or shallow microbial profiling, with limited integration of metagenomic and metabolomic analyses to elucidate underlying mechanisms. In particular, it remains unclear how specific probiotic strains influence patient-reported quality of life through defined shifts in microbial composition and metabolite production.

*Bifidobacterium animal* subsp. *lactis* V9 (V9) is a well-characterized probiotic strain originally isolated from the gut microbiota of healthy individuals. It exhibits robust acid and bile tolerance, strong adhesion to intestinal epithelium, potent antioxidant activity, and immunomodulatory properties. Notably, V9 has been shown to enhance intestinal barrier integrity and modulate microbial metabolites, including short-chain fatty acids (SCFAs) [29]. In preclinical and limited clinical studies, V9 supplementation has been associated with improvements in functional gastrointestinal disorders such as constipation and reductions in inflammatory markers. These effects appear to coincide with alterations in gut microbial composition, attenuation of inflammatory signaling pathways, and modulation of host metabolic profiles [29–31]. Although these findings suggest that microbiota reshaping may contribute to mucosal homeostasis, direct evidence demonstrating that microbial modulation alone drives epithelial repair remains limited. Therefore, the potential mucosal benefits of V9 are likely multifactorial, involving both microbiota-dependent and host-mediated mechanisms. In preclinical and clinical settings, V9 supplementation has alleviated functional gastrointestinal disorders such as constipation and reduced inflammation by reshaping the gut microbiota, dampening inflammatory signaling, and reprogramming metabolic pathways [29–31]. *Bifidobacterium animalis* subsp. *lactis* has been included in the List of Microorganisms Permitted for Use in Food issued by the National Health Commission of China since 2010, and strains from this species have been widely applied in probiotic foods and dietary supplements. These attributes position V9 as a compelling candidate for adjuvant intervention in patients undergoing intensive chemotherapy for advanced gastrointestinal cancers.

Building on this rationale, the present study tests the hypothesis that V9 supplementation during chemotherapy improves quality of life in patients with advanced gastric or esophageal cancer by restoring gut microbial balance and modulating bioactive metabolite profiles. Using a randomized, double-blind, placebo-controlled design integrated with longitudinal metagenomic and metabolomic profiling, this work seeks to move beyond symptomatic assessment toward a mechanistic understanding of how a defined probiotic intervention influences the host–microbiota–metabolite axis. The findings aim to provide evidence-based support for the use of V9 as a safe and effective adjunctive therapy to enhance resilience, reduce treatment-related toxicity, and improve overall well-being in this vulnerable patient population.

## Materials and methods

### Experimental design

This randomized, double-blind, placebo-controlled trial was conducted over 18 weeks between April 2024 to March 2025 at Beijing Shijitan Hospital (affiliated with Capital Medical University) and Sichuan Cancer Hospital. Initially, 120 patients with histologically or cytologically confirmed advanced (stage IV) gastric and esophageal cancer were screened for eligibility. Exclusions occurred due to disease progression leading to death, discontinuation of chemotherapy, elective surgery, voluntary withdrawal, or transfer to other facility. Ultimately, 104 eligible participants were enrolled and randomly assigned to either the probiotic group (Pro group; *n* = 51) or the placebo group (Pla group; *n* = 53).

**Figure 1:**
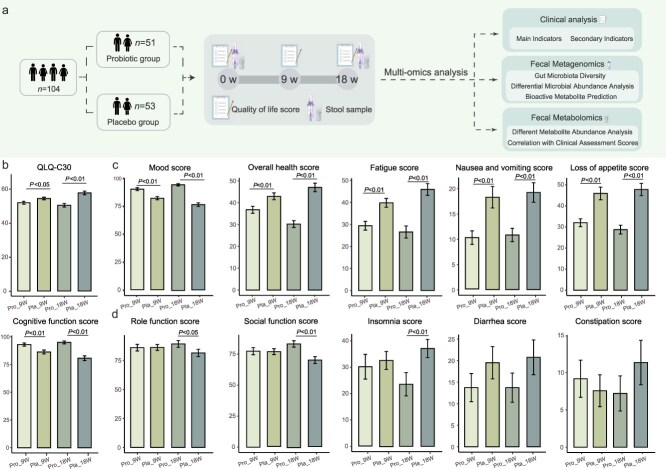
Study design and effects of *B. animalis* subsp. *lactis* V9 supplementation on quality of life in patients with advanced gastric or esophageal cancer. (a) Workflow of the randomized, double-blind, placebo-controlled trial, including clinical assessments, stool collection, and multi-omics analyses (metagenomics, and metabolomics) at baseline (week 0) and post-intervention (Week 18). Pla = placebo group; Pro = probiotic group. (b) Total EORTC QLQ-C30 scores at week 9, and week 18. (c, d) Domain scores for (c) mood, overall health scores, fatigue, nausea and vomiting, appetite loss, cognitive function; (d) role function, social function, insomnia, diarrhea, and constipation at Weeks 9 and 18. Error bars represent standard error of the mean (SEM), and *P* < .05 was considered statistically significant.

At enrollment, trained clinical nutritionists or clinical nutrition nurses, standardized by the project team, guided participants in completing the case report form. Follow-up assessments were conducted at Weeks 9 and 18 to update clinical and quality-of-life data. Concurrently, a stool samples were collected from all participants at baseline (Week 0) and postintervention (Week 18) for downstream microbiome and metabolome analyses (Fig. 1a).

Both the placebo and the active intervention, V9 (Kex02), were supplied as identical-appearing dry powders by Zhejiang Jinhua Yinhe Biotechnology Co., Ltd., China, each 2 g sachet containing 1 × 10^10^ colony-forming units. A dedicated study coordinator managed storage, inventory, and distribution. Participants were instructed to consume two doses daily (totaling 4 g/day) for 18 consecutive weeks.

### Inclusion and exclusion criteria

#### Inclusion criteria

Participants were required to me*et al*l of the following: (i) histological or cytological diagnosis of stage IV gastric or esophageal cancer; (ii) age between 18 and 80 years; (iii) Eastern Cooperative Oncology Group performance status score of 0–2; (iv) expected survival exceeding 4 months; (v) scheduled to receive systemic chemotherapy; (vi) ability to maintain oral intake; and (vii) provision of written informed consent with willingness and capacity to adhere to all study procedures, including scheduled visits, interventions, and assessments.

#### Exclusion criteria

Individuals were excluded if they: (i) had active infections, psychiatric disorders, significant cardiovascular or cerebrovascular diseases, severe cardiopulmonary impairment, or other conditions deemed incompatible with study participation by the investigator; (ii) had received multiple prior chemotherapy regimens or extensive radiotherapy, or had comorbidities such as bone marrow metastases, severe infections, adrenal insufficiency, or major complications; (iii) were pregnant; (iv) were unable to adhere to the intervention schedule or complete required procedures; (v) had gastrointestinal contraindications (e.g. oral intake intolerance, intestinal obstruction, active intestinal bleeding, severe short bowel syndrome, or high-output enterocutaneous fistula); (vi) were concurrently enrolled in another interventional clinical trial; (vii) exhibited severe comorbidities (e.g. uncontrolled heart disease, diabetes, hyperlipidemia, electrolyte imbalance, or abnormal liver/kidney function) judged unsuitable by the investigator; or (viii) had active autoimmune disease or a history of autoimmune disease with anticipated relapse.

### Ethical approval

This research protocol has been approved by the Ethics Committee of Beijing Shijitan Hospital affiliated with Capital Medical University (Approval No.: IIT2023-006-004). All procedures were conducted in accordance with the ethical principles of the Declaration of Helsinki. All participants signed informed consent forms before enrollment.

### Outcome assessment

The primary endpoint was health-related quality of life, assessed using the European Organisation for Research and Treatment of Cancer Quality of Life Questionnaire Core 30 (EORTC QLQ-C30) [19], which is applicable to various cancer types and treatment stages and provides comprehensive and systematic patient-reported quality-of-life data. The EORTC QLQ-C30 scale consists of 30 items divided into 15 dimensions, including 5 functional dimensions (physical functioning, role functioning, mood functioning, social functioning, and cognitive functioning), 3 symptom dimensions (fatigue, pain, and nausea/vomiting), 6 single items (dyspnea, appetite loss, insomnia, constipation, diarrhea, and financial difficulties), and 1 overall quality-of-life dimension. Only the overall quality of life scale uses a 1–7-point scale, while all other items use a 4-point scale (“not at all” 1 point, “a little” 2 points, “quite a bit” 3 points, “very much” 4 points). All questionnaires were self-administered by participants under standardized instructions. Trained healthcare personnel were present to provide procedural guidance only (e.g. clarification of response format or checking for missing items) without interpreting questionnaire content or influencing responses. Participants completed the questionnaires independently in a quiet clinical setting. The same standardized procedure was applied to both groups, and study staff were blinded to group allocation during data collection.

Secondary endpoints included changes in chemotherapy-related gastrointestinal toxicities, such as diarrhea, constipation, nausea, vomiting, and appetite loss, the severity of which was assessed using relevant items from the EORTC QLQ-C30 scale.

Fecal samples were collected at baseline and Week 18 at the hospital or at home using sterile sampling tubes containing DNA preservative solution (Guangdong Longsee Biomedical Co., Ltd., Guangzhou, China), per manufacturer instructions. Collected samples were immediately placed in a −18°C freezing environment for temporary storage and then transferred to a −80°C ultra-low temperature freezer for long-term storage using liquid nitrogen transport containers within 2 h, to facilitate subsequent metagenomic and metabolomics analyses.

### Metagenomic sequencing, quality control, and functional prediction

Metagenomic DNA was extracted from baseline and postintervention fecal samples using a commercial fecal DNA extraction kit (DP712, Tiangen Biotech, Beijing, China). Libraries were constructed using the NEBNext® Ultra DNA Library Construction Kit (E7370S/L, New England Biolabs, Ipswich, MA, USA) and sequenced on the Illumina NovaSeq 6000 platform (Novogene Technology Co., Ltd., Beijing, China), generating 1.45 Tbp of high-quality data (mean = 7.90 Gbp/sample). It is worth noting that some participants withdrew from the study midway, or were unable to successfully collect stool samples at all time points, and a small number of samples were excluded due to failure to meet sequencing or quality control standards. Therefore, a total of 183 successfully sequenced samples were obtained (Table S1).

Raw reads were assembled into contigs (≥2000 bp) using MEGAHIT [32]. Metagenome-assembled genomes (MAGs) were reconstructed via MetaBAT2 [32] and DAS Tool [33], with default settings, then merged across samples using an in-house script. The quality of MAGs was assessed with CheckM [34]; only high-quality MAGs (completeness ≥80%, contamination ≤5%) were retained. Species-level genome bins (SGBs) were generated by dereplicating MAGs with dRep [35] (parameters -pa 0.95, -sa 0.95) (Table S2). Relative abundances of SGBs were quantified using CoverM (https://github.com/wwood/CoverM) with alignment identity ≥95% and minimum covered fraction ≥40% [-min-read-percent-identity 0.95 -min-covered-fraction 0.4].

Functional potential was inferred by annotating SGB-encoded open reading frames against the Kyoto Encyclopedia of Genes and Genomes (KEGG) database. Gut-associated metabolic modules (GMMs) were predicted using Omixer-RPM (https://github.com/raeslab/omixer-rpm) with a module coverage threshold of 66% (-c 0.66), integrating KEGG ortholog profiles to assess pathway completeness in accordance with MetaCyc-based approaches [24, 25]. This enabled identification of biologically relevant metabolic pathways encoded by high-quality SGBs in the context of host–microbiota interactions. Finally, the classification distribution of gut bioactive metabolite features in high-quality sequences was determined using the MelonnPan-predict workflow [36].

### Fecal metabolite profiling by liquid chromatography–mass spectrometry

Fecal samples were thawed, concentrated, and dried at 4°C. An appropriate amount of the dried sample was placed in a 2 ml centrifuge tube, and 600 μl of 80% methanol solution containing 4 μg/ml of 2-chloro-L-phenylalanine (internal standard) was added. The mixture was mixed for 30 s. The mixture was then ground, sonicated, and centrifuged at 12 000 rpm for 10 min at 4°C. LC-MS analysis was performed using an ACQUITY UPLC HSS T3 column (2.1 × 100 mm, 1.8 μm) maintained at 40°C. The flow rate was 0.3 mL/min, and the injection volume was 5 μl. The mobile phases consisted of acetonitrile/water with 0.1% formic acid for positive ion mode, and acetonitrile/water with 5 mM ammonium formate for negative ion mode.

The supernatant was collected, filtered through a 0.22 μm filter membrane, and used for LC-MS analysis. Chromatographic separation was performed using an Agilent 1290 Infinity ultra-high performance liquid chromatography system (Agilent Technologies, Santa Clara, California, USA), equipped with an ACQUITY UPLC BEH amide column (100 × 2.1 mm, 1.7 μm; Waters Corporation, Milford, MA, USA). Gradient elution of the analytes was performed using a mixture of an aqueous solution containing 25 mM ammonium acetate and 25 mM ammonia with acetonitrile. Each sample was analyzed under electrospray ionization conditions in both positive and negative ionization modes.

Raw data were converted and processed using ProteoWizard and the R package XCMS. Principal component analysis and partial least squares discriminant analysis were applied to identify candidate metabolites differentiating the Pro and Pla groups (significance thresholds: variable importance in projection [VIP] > 2 and *P* < .05). A VIP value > 1.0 is generally regarded as significant for group discrimination, and thresholds of VIP > 1.5 or VIP > 2 have been adopted in previous studies. In this study, a screening threshold of VIP > 2 and *P* < .05 was adopted to obtain a higher-confidence set of core differential metabolites [37]. Putative metabolite identities were assigned by matching *m/z* and retention time against the HMDB (http://www.hmdb.ca/), METLIN (http://metlin.scripps.edu/), Massbank (http://www.massbank.jp), and KEGG (http://www.kegg.com/) databases. Identified metabolites were further mapped to biochemical pathways using KEGG and MetaCyc to contextualize their roles in microbial and host metabolic networks.

### Statistical analysis

All analyses were performed in R (v4.4.2); figures were refined using Adobe Illustrator. Continuous data are reported as mean ± standard deviation. Baseline demographic differences (age, sex) were evaluated using chi-square tests. Between-group comparisons of continuous outcomes were achieved with Wilcoxon rank-sum tests, with *P*-values adjusted for multiple testing using the Benjamini–Hochberg procedure.

Microbiome and metabolome data were visualized via principal coordinates analysis using the vegan, optparse, mixOmics, ggplot2, and ggpubr R packages. Statistical significance in multivariate space was assessed by permutational multivariate analysis of variance (PERMANOVA) with 999 permutations (Adonis test). A *P-*value <.05 was considered statistically significant. Associations among quality-of-life scores, microbial taxa, and fecal metabolites were quantified using Spearman correlation coefficients.

## Results

### Patient baseline characteristics

Study design and participant demographics are summarized in Fig. 1a and Table S3. The Pla group (*n* = 53) had a mean age of 61.11 ± 13.36 years and a male-to-female ratio of 35:18. The Pro group (*n* = 51) had a mean age of 58.23 ± 10.48 years and a male-to-female ratio of 29:22. Mean body mass index was comparable between groups (Pla: 22.0 ± 3.4 kg/m^2^; Pro: 21.9 ± 3.4 kg/m^2^). Statistical comparison revealed no significant differences between the two groups in age, sex, body mass index, smoking or alcohol use history, and baseline blood parameters, including total protein, albumin, white blood cell count, neutrophils, lymphocytes, platelets, and tumor markers (CA199, CA125, carcinoembryonic antigen, and alpha-fetoantigen) (*P* > .05; Table S4), confirming balanced randomization. Information on patients’ comorbidities is provided in Table S3.

### Supplementation of V9 improved clinical symptoms and quality of life

Patients in the Pro group exhibited significantly lower EORTC QLQ-C30 total scores, indicating better overall quality of life, compared to the Pla group at both Week 9 (Pro: 51.8 ± 6.1 vs. Pla: 54.3 ± 6.1) and Week 18 (Pro: 50.4 ± 7.4 vs. Pla: 57.6 ± 7.3; *P* < .01; Fig. 1b). Further domain-level analysis revealed that V9 supplementation significantly improved mood, overall health status, fatigue, nausea and vomiting, appetite loss, and cognitive functioning at both time points (*P* < .01; Fig. 1c).

Improvements in role functioning, social functioning, and insomnia reached statistical significance only Week 18 (*P* < .01; Fig. 1d). Although diarrhea and constipation scores showed a decreasing trend in the Pro group by Week 18, these changes did not reach statistical significance. No significant differences were observed between groups for pain, dyspnea, or financial difficulties throughout the intervention period (Table S5). Overall, these findings indicate that V9 supplementation conferred sustained, multidimensional benefits to quality of life, while certain symptom domains remained unaffected.

### Supplementation of V9 modulated gut microbiota composition and metabolic potential

**Figure 2:**
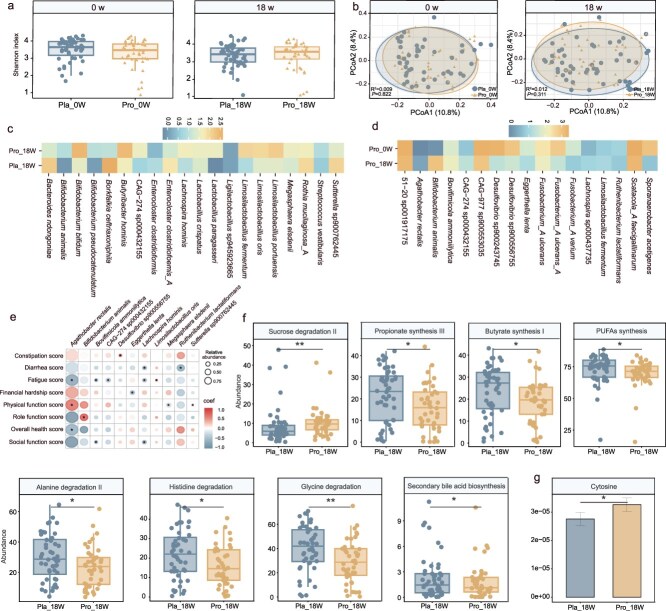
V9 intervention resulted in changes in gut microbiota composition, gut metabolic modules (GMMs), and predicted metabolic changes. (a) α-Diversity (Shannon index) and (b) β-diversity (principal coordinates analysis based on Bray–Curtis distance, with Adonis test results) of fecal microbiota in the placebo (Pla) and probiotic (Pro) groups at baseline and Week 18. Box plots display median (center line), interquartile range (box), and 1.5× interquartile range (whiskers). (c) Heatmap of species-level genome bins (SGBs) with significantly different relative abundances between the Pla and Pro groups at Week 18; and (d) longitudinal changes in SGB abundance within the Pro group from baseline to week 18 (*P* < .05, Wilcoxon rank-sum test). The scale represents relative abundance. (e) Correlation between differentially abundant SGBs and EORTC QLQ-C30 domain scores in the Pro group at Week 18. Circles denote negative and positive correlations, respectively; circle size reflects SGB relative abundance. (f) Responsive GMMs showing differences before and after probiotic intervention. (g) Predicted levels of differential gut bioactive metabolites before and after V9 intervention, inferred from metagenomic data using MelonnPan. Error bars represent standard deviation statistical differences were evaluated using the Wilcoxon rank-sum test, with *P* < .05 considered significance.

Metagenomic profiling of stool samples from all 104 participants was performed at baseline and postintervention. Alpha diversity (Shannon index) and beta diversity (principal coordinates analysis based on Bray–Curtis dissimilarity) showed no significant differences between groups or over time (Fig. 2a and b; Adonis *P* > .05), suggesting that V9 did not broadly alter the global structure of the gut microbiota.

To detect more subtle, taxon-specific shifts, we focused on SGBs with mean relative abundance ≥0.01% and no baseline differences between groups. At Week 18, 20 SGBs exhibited significant differential abundance (*P* < .05; Fig. 2c, Table S6). Notably, the Pro group showed increased abundance of beneficial taxa, including *Limosilactobacillus fermentum*, *Megasphaera elsdenii*, *L. portuensis*, *B. pseudocatenulatum*, and *B. bifidum*. In contrast, several taxa that have been associated with inflammatory conditions or opportunistic infections, such as *Bacteroides ndongoniae* [38], *Borkfalkia ceftriaxoniphila* [38], *Sutterella* sp900762445 [39], and *Enterocloster clostridioformis* [40] was significantly depleted.

Longitudinal analysis revealed consistent temporal trends: SCFA-producing bacteria, including *B. animalis*, *A. rectalis*, *Lachnospira* sp000437735, and *L. fermentum*, progressively increased in relative abundance over the 18-week intervention. In contrast, *Sporanaerobacter acetigenes*, *Fusobacterium varium*, and *Ruthenibacterium lactatiformans* showed a significant decline (*P* < .05; Fig. 2d, Table S6).

To link microbial shifts to clinical outcomes, we examined associations between species-level abundances and EORTC QLQ-C30 domain scores using multivariable modeling (MaAsLin2). After adjustment for potential confounders, *B. animalis* was significantly negatively correlated with role functioning score (coef = −0.96, *P* = .03). *Lachnospira hominis* showed significant negative correlations with fatigue (coef = −0.48, *P* = .03), social functioning (coef = −0.59, *P* = .006), and diarrhea (coef = −0.57, *P* = .005). *Agathobacter rectalis* was positively correlated with overall health (coef = 0.69, *P* = .033) but negatively correlated with fatigue (coef = −0.79, *P* = .015) and physical functioning (coef = −0.99, *P* = .01). *Ruthenibacterium lactatiformans* was also negatively correlated with diarrhea score (coef = −0.71, *P* = .009; Fig. 2e). In contrast, unadjusted Spearman correlation analysis did not yield statistically significant associations for these taxa.

### Probiotics regulate gut metabolic modules and predict gut bioactive compounds

This study systematically analyzed the regulatory effects of specific probiotics on gut metabolic modules (GMMs). Metabolic reconstruction based on metagenomic data, combined with the MetaCyc and KEGG databases, assessed changes in 606 SGB-encoded GMMs. The identified functional modules originated from 11 bacterial phyla, with Bacillota being the most prevalent (67.4%), followed by Bacteroidota (15.8%) and Pseudomonadota (7.2%). Comparative analysis of the cumulative abundance of SGB-encoded GMMs after intervention revealed that V9 intake significantly enriched the sucrose degradation II pathway while significantly reducing the abundance of multiple metabolic-related pathways, including propionic acid synthesis III, butyrate synthesis, polyunsaturated fatty acid biosynthesis, and various amino acid degradation pathways (such as alanine degradation II, histidine degradation, and glycine degradation) (*P* < .05; Fig. 2f). Notably, no significant differences were observed between the two groups in the secondary bile acid biosynthesis pathway related to bile acid metabolism (*P* > .05; Fig. 2f; Table S7). The gut microbiota–mediated bioactive metabolite profile was further inferred using the MelonnPan-predict workflow. In the Pro group, the predicted abundance of cytosine was significantly increased after 18 weeks of V9 supplementation (*P* < .05; Fig. 2g; Table S7). These results suggest that V9 intervention may promote improved host metabolic homeostasis by reprogramming the metabolic functions of the gut microbiota and modulating the metabolite profile.

### Fecal metabolome changes induced by V9 supplementation

**Figure 3:**
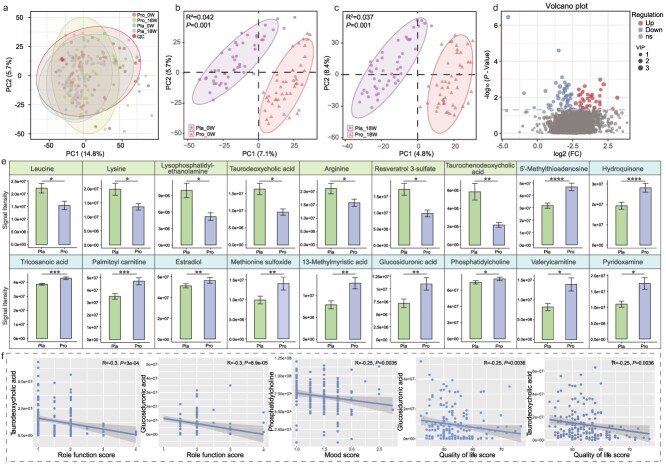
Fecal metabolomic profiling reveals V9-induced metabolic remodeling. (a) Principal component analysis and (b, c) partial least squares discriminant analysis score plots comparing fecal metabolite profiles between placebo (Pla) and probiotic (Pro) groups at baseline and Week 18. Adonis *P*-values are shown. (d) Volcano plot of differentially abundant metabolites at Week 18 (Pro vs. Pla). The *x*-axis shows fold change (log2 ratio scale), and the *y*-axis shows the negative log10 of *P*-values (a higher value represents a stronger statistical significance). (e) Relative abundance of selected differential metabolites. Error bars represent standard deviation, *P* < .05, ^*^P < .01, ^**^P < .001, ^***^P < .0001. (Wilcoxon rank-sum test). (f) Spearman correlation between key differential metabolites and EORTC QLQ-C30 domain scores. The gray line indicates the regression fit; the shaded area represents the 95% confidence interval.

Principal component analysis of quality control samples demonstrated tight clustering in the score plot (Fig. 3a), confirming system stability and reproducible chromatographic performance throughout the LC-MS run. Partial least squares discriminant analysis revealed a clear separation in fecal metabolite profiles between the Pro and Pla groups after 18 weeks of intervention (*R*^2^ = 0.037, *P* = .001; Fig. 3b and c). To minimize potential batch effects, all fecal samples were analyzed within a single analytical batch using a randomized injection order. Quality control (QC) samples were injected at regular intervals throughout the run to monitor system stability and analytical reproducibility.

Volcano plot analysis identified 4261 metabolites with differential abundance between groups. To reduce false positives, we applied multiple selection thresholds, including a Wilcoxon rank-sum test (*P* < .05), a fold change (FC ≥ 1.5 or ≤ 0.67), and a variable importance in projection (VIP ≥ 1.0), 69 metabolites were identified as significantly altered after the intervention (30 increased and 39 decreased; Fig. 3d). To enhance biological interpretability and reduce potential baseline bias, we further prioritized metabolites that showed no significant difference between groups at baseline but became significantly different after the intervention, as these were more likely to reflect intervention-associated metabolic changes. Following annotation in both positive and negative ionization modes, 18 metabolites m*et al*l selection criteria and were retained as core differential metabolites (Fig. 3e, Table S8). Functionally, these metabolites mainly belonged to several biochemical classes, including amino acids, lipid-related metabolites, bile acid derivatives, and nucleotide-related compounds, suggesting broad metabolic remodeling following V9 supplementation. For example, in the Pro group, levels of taurochenodeoxycholic acid, several amino acids (leucine, lysine, arginine), and lysophosphatidylethanolamine were significantly reduced, whereas 5′-methylthioadenosine, hydroquinone, triicosanic acid, palmitoylcarnitine, pyridoxamine, 13-methylmyristic acid, and phosphatidylcholine were elevated.

To evaluate the clinical relevance of these metabolic shifts, Spearman correlation analysis was performed between key metabolites and quality-of-life domains (Fig. 3f). Taurodeoxycholic acid and glucuronic acid were both negatively correlated with role functioning (*r* = −0.30, *P* < .001 for both). Phosphatidylcholine showed a significant negative association with emotional functioning (*r* = −0.25, *P* = .0035). Additionally, both glucuronic acid and taurdeoxycholic acid were negatively correlated with quality-of-life scores (*r* = −0.25, *P* = .0036 for both). These results suggest that V9 may alleviate chemotherapy-related symptoms and enhance quality of life, in part, by reshaping the gut metabolome toward a more favorable metabolic state.

### Integrative analysis of microbiota, metabolites, and clinical indicators

**Figure 4:**
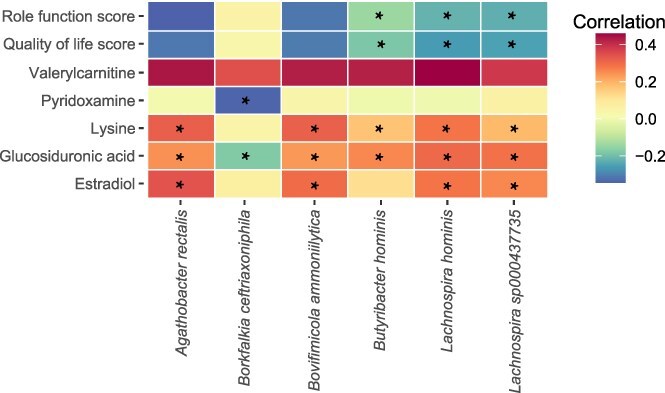
Heatmap of the correlation between microbiota, metabolites, and quality of life in patients with gastrointestinal cancer. Integrative correlation heatmap showing associations among core microbial taxa, key differential metabolites, and quality-of-life indicators. Spearman correlation coefficients are displayed with corresponding significance levels.

To further explore the relationships among gut microbiota, metabolites, and clinical outcomes, we performed an integrative correlation analysis of core bacterial taxa, key metabolites, and quality-of-life indicators (Fig. 4, Table S9). *Agathobacter rectalis* and *Bovifimicola ammoniilytica* were negatively correlated with role functioning and overall quality-of-life scores, while showing significant positive correlations with lysine and valerylcarnitine. In addition, *B. ceftriaxoniphila* was negatively associated with pyridoxamine but positively correlated with valerylcarnitine. Notably, valerylcarnitine showed strong positive correlations with multiple taxa, including *Lachnospira* sp000437735, *Butyribacter hominis*, and *A. rectalis*. Collectively, these results suggest coordinated relationships among gut microbes, metabolic profiles, and clinical indicators.

## Discussion

**Figure 5:**
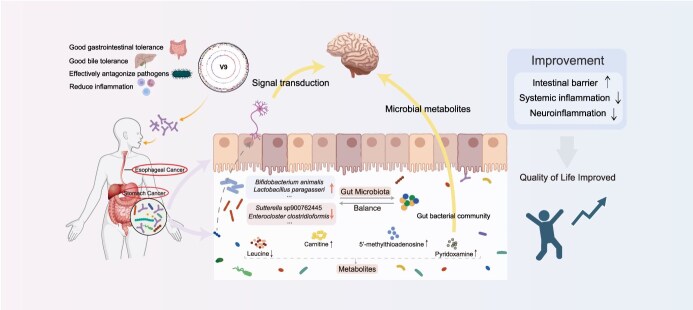
Proposed mechanistic model of *B. animalis* subsp. *lactis* V9 action during chemotherapy. *Bifidobacterium animalis* subsp. *lactis* V9 supplementation enriches beneficial bacteria (e.g. *B. animalis*, *A. rectalis*, *L. hominis*) and depletes pathobionts (e.g. *F. varium* and *E. clostridioformis*), leading to favorable shifts in microbial metabolites, including increased pyridoxamine and palmitoylcarnitine and decreased taurine-conjugated bile acids. These changes enhance intestinal barrier integrity, reduce inflammation, and modulate the gut–brain axis, collectively alleviating gastrointestinal symptoms, fatigue, and psychological distress, thereby improving overall quality of life in chemotherapy-treated cancer patients.

This randomized, double-blind, placebo-controlled trial demonstrates that supplementation with V9 during chemotherapy significantly improves quality of life in patients with advanced gastric or esophageal cancer. The findings support the central hypothesis that V9 exerts its benefits not through broad restructuring of the gut ecosystem, but via precise modulation of specific microbial taxa and their metabolic outputs, thereby linking the gut microbiota–metabolite axis to patient-centered clinical outcomes (Fig. 5).

Chemotherapy, while essential in oncology, frequently induces debilitating gastrointestinal and systemic toxicities, including nausea, vomiting, fatigue, appetite loss, and cognitive dysfunction, that compromise treatment adherence and patient well-being. Although antiemetics and standard supportive care are routinely used, residual symptoms remain a significant clinical challenge. Probiotics have emerged as a promising adjuvant strategy, with prior studies reporting benefits in conditions such as functional dyspepsia, chemotherapy-associated diarrhea, and impaired mucosal integrity [41, 42]. These effects are thought to arise through multiple interconnected mechanisms, including enhancement of the intestinal barrier, immune modulation, and stabilization of the gut microbiota [43, 44]. Importantly, probiotic actions extend beyond the gut lumen via a multidimensional network involving brain–gut axis regulation, inflammatory and immune signaling, and neuroendocrine and vagal pathways [45, 46], positioning them as potential modulators of both physical and psychological dimensions of quality of life during cancer therapy. However, evidence linking specific probiotic strains to multi-omic shifts and holistic quality-of-life improvements in cancer patients has been scarce. This study bridges that gap by integrating clinical phenotyping with deep metagenomic and metabolomic profiling.

Our study found that the adjunctive V9 supplementation significantly improved EORTC QLQ-C30 scores across emotional, cognitive, social, and role functioning domains, with sustained effects through Week 18, consistent with a prior work demonstrating the improvement of bowel symptoms and quality of life in colorectal cancer survivors [47]. To facilitate interpretation of these quality-of-life outcomes, normative data from healthy populations reported in previous studies were used as a reference. In general, healthy individuals exhibit relatively high scores in functional domains (~70–90 points) and low scores in symptom domains on the EORTC QLQ-C30 [48]. In the present study, functional scores increased following V9 intervention and approached or even exceeded the levels reported in healthy populations in several domains, suggesting a meaningful improvement in functional well-being. Meanwhile, symptom scores, including fatigue, nausea/vomiting, appetite loss, insomnia, and diarrhea, generally decreased after the intervention, indicating an overall improvement in symptom burden, although some domains remained different from normative population values. Compared with the normative values reported for the general population, these findings suggest that V9 intervention may contribute to improvements in both functional status and symptom burden in cancer patients. Notably, while diarrhea and constipation showed a favorable trend in the probiotic group, the difference did not reach statistical significance, a finding also reported previously [49]. This may reflect the cyclical nature of chemotherapy-induced gastrointestinal toxicity, which peaks 1–2 days post-treatment. The case report form used in this study capture symptom burden at fixed intervals (Weeks 9 and 18) but may have missed acute fluctuations, potentially underestimating the impact of V9 on transit-related symptoms. Future studies employing daily or cycle-synchronized symptom tracking could better capture these dynamics.

Metagenomic analysis revealed no significant changes in α- or β-diversity, indicating that the clinical benefits of V9 arise not from broad microbiota restructuring but from precise, species-level modulation. The probiotic group exhibited consistent enrichment of beneficial taxa, including *B. pseudocatenulatum*, *B. animalis*, *B. bifidum*, *L. fermentum*, and *L. portuensis*, many of which or closely related species are known for mucosal adhesion, SCFA production, gut barrier enhancement, and immunomodulation [50–53]. Notably, V9 significantly increased the abundance of butyrate-producers such as *A. rectalis*, *B. hominis*, and *L. hominis*. Butyrate, plays a well-established role in maintaining intestinal homeostasis by alleviating mucosal damage, reducing inflammation, regulating appetite, and relieving diarrhea, symptoms commonly exacerbated by chemotherapy [54–57]. Insufficient butyrate production has also been linked to fatigue cognitive impairment, and sleep disturbances [58]. In our multivariable association analysis, *A. rectalis* abundance was associated with improved global health scores and reduced fatigue and cognitive dysfunction. Although unadjusted correlation analysis did not reach statistical significance, the observed trends are biologically plausible and consistent with previous reports [59, 60].

Concurrently, V9 significantly suppressed several opportunistic or pro-tumorigenic taxa. *Enterocloster clostridioformis* (associated with bacteremia and intestinal dysfunction [61]), *Eggerthella lenta* (which activates the RHEB/mTOR/AKT pathway to promote cancer progression [62, 63]), and *F. varium* (a driver of colorectal carcinogenesis, chemoresistance, and immunosuppression [64–66]) were all markedly reduced. The depletion of these pathobionts, coupled with the enrichment of beneficial SCFA producers, suggests that V9 fosters a gut ecosystem more conducive to treatment tolerance and overall well-being during chemotherapy.

This study explored the association between probiotic intervention and changes in the metabolic potential of the gut microbiota, analyzing the relationship at the functional module level. Increased abundance of the sucrose degradation pathway suggests enhanced microbial utilization of carbohydrates, an enhancement typically associated with the production of beneficial metabolites. Conversely, decreased abundance of the amino acid degradation pathway may indicate a decline in microbial amino acid catabolism, which previous studies have shown is associated with reduced production of potentially harmful metabolites such as ammonia, amines, and phenols [67, 68]. Notably, some pathways associated with SCFA synthesis also showed a downward trend, suggesting that the metabolic shift induced by probiotic intervention is unlikely to be a simple unidirectional enhancement but rather reflects a more complex metabolic reprogramming. This pattern may be related to reduced substrate availability [69, 70]. Furthermore, the reduction in polyunsaturated fatty acid (PUFA) biosynthesis pathways may reflect a broader adjustment in lipid metabolism, which may, in turn, influence the production of inflammation-associated lipid mediators and host immune responses [71]. Conversely, no significant changes were observed in pathways associated with secondary bile acid biosynthesis, suggesting that bile acid metabolism may not be the primary contributor to the observed effects. Furthermore, metabolite inference based on MelonnPan suggests that the probiotic group has higher predicted cytosine levels, which may reflect increased microbial turnover or biosynthetic activity, processes associated with mucosal repair and cell regeneration [72] Overall, V9 supplementation is associated with the remodeling of gut microbiota metabolic function, and these functional changes may collectively contribute to the improvement of host health.

Metabolomic profiling further substantiated these microbial shifts. Supplementation with V9 significantly increased fecal levels of pyridoxamine (vitamin B6), 5′-methylthioadenosine (MTA), palmitoylcarnitine, and 13-methylmyristic acid while decreasing taurochenodeoxycholic acid, lysophosphatidylethanolamine, and several amino acids (leucine, lysine, arginine). Pyridoxamine is essential for neurotransmitter synthesis and energy metabolism; it is absorbed in the intestine, converted to pyridoxal in the liver, and subsequently taken up by the brain, where it is phosphorylated to pyridoxal phosphate, a critical coenzyme for the synthesis of neurotransmitters such as dopamine and serotonin, and gamma-aminobutyric acid (GABA). The elevation of fecal pyridoxamine in the V9 group likely reflects increased colonic production, which, following absorption and hepatic conversion, may support central nervous system neurotransmitter synthesis and contribute to the observed improvements in cognitive function, mood, and fatigue reduction [73–75]. MTA is a natural polyamine derivative produced by Bifidobacteria through methionine metabolism, and it possesses anti-inflammatory, anti-angiogenic, and pro-apoptotic properties [76]. Notably, previous studies have shown that MTA can enhance intestinal mucosal barrier function by promoting the expression of tight junction proteins (including ZO-1, Claudin, and Occludin) and significantly reduce intestinal tissue damage and disease activity in mice with dextran sulfate sodium–induced colitis through its anti-inflammatory effects [77]. The elevation in the V9 group suggests a possible role in modulating the tumor microenvironment, although whether this translates into direct antitumor effects in patients remains to be investigated. Palmitoylcarnitine supports mitochondrial β-oxidation and cellular energy homeostasis [31, 78], potentially countering chemotherapy-induced metabolic exhaustion. Additionally, 13-methylmyristic acid, a branched-chain fatty acid with documented antitumor and anti-inflammatory properties [79], was significantly elevated in the V9 group. This finding raises the possibility that V9 may contribute to a tumor-suppressive metabolic environment, though further studies are needed to confirm its functional relevance in patients. Critically, the reduction in taurine-conjugated bile acids aligns with the ability of V9 to suppress bile acid-transforming pathobionts; excessive secondary bile acids are known to disrupt barrier function, promote inflammation, and drive nausea and diarrhea via the gut–liver axis [80]. In the gut–brain axis, the reduction and “normalization” of secondary bile acids may alleviate neuroinflammatory burden associated with overactivation of TGR5/FXR, restore blood–brain barrier integrity, and optimize gut–brain signaling, thereby partially explaining the observed improvements in mood, cognitive function, and sleep [81]. Since the gut microbiota is responsible for the biotransformation of bile acids, changes in bile acid metabolism may affect the integrity of the epithelial barrier and the homeostasis of the mucus layer, linking microbial metabolic activity to gut mucosal health [82]. The negative correlation between taurodeoxycholic acid and quality-of-life scores further underscores the clinical relevance of this metabolic shift.

Additionally, phosphatidylcholine levels were significantly elevated in the V9 group compared to placebo. As a major phospholipid constituent of neuronal membranes and a precursor for neurotransmitter synthesis, phosphatidylcholine plays a critical role in cognitive and emotional health; its deficiency has been linked to depression severity [83, 84]. Moreover, phosphatidylcholine is a core component of the intestinal mucus layer, where it contributes to mucosal barrier integrity and protection against luminal stressors [85, 86]. The V9-induced increase in fecal phosphatidylcholine may therefore support both neuroprotective and mucosal-repair functions, limiting the translocation of pro-inflammatory microbial products, thereby reducing systemic and neuroinflammation while creating a favorable environment for beneficial metabolites to enter the circulation. This may contribute to the observed improvements in emotional well-being, cognitive functioning, and gastrointestinal resilience during chemotherapy.

Together, these microbial and metabolic changes likely converge on the gut–brain axis to drive multidimensional improvements. By fortifying the intestinal barrier and reducing systemic inflammatory cues, V9 may dampen neuroinflammation and visceral hypersensitivity. Simultaneously, metabolite-mediated support of neuronal and energetic function (via B6, carnitine, and 5′-methylthioadenosine) may enhance cognitive resilience and mood. The observed improvements in insomnia, social functioning, and emotional well-being thus reflect a systems-level restoration of host–microbiota equilibrium. Compared with other probiotics, V9 exhibits a distinctive “precision regulation” pattern: it selectively enriches beneficial bacterial species (e.g. *A. rectalis*, *L. hominis*, *B. pseudocatenulatum*) while effectively inhibiting opportunistic pathogens (e.g. *F. varium*, *E. clostridioformis*, *E. lenta*), without significantly altering gut microbial α-diversity or β-diversity. In contrast, many commercial probiotics, particularly multi-strain formulations are designed to exert broad-spectrum effects and often lack targeted inhibitory capacity against specific pathobionts [87]. Furthermore, V9-induced metabolic reprogramming is characterized by a coordinated shift involving concurrent elevation of pyridoxamine, 5′-methylthioadenosine, and palmitoylcarnitine, along with a marked reduction in taurine-conjugated bile acids. While a probiotic formulation containing *L. acidophilus* NCFM and *B. lactis* Bl-04 has been reported to induce epigenetic modifications, it did not elicit comparable alterations in bile acid profiles [88]. Notably, V9 may exert more targeted effects on the gut–brain axis compared to strains such as *L. acidophilus* or *B. longum*, whose mechanisms are primarily confined to the local intestinal environment. V9 establishes mechanistic links through its specific metabolites, for instance, the conversion of pyridoxamine to pyridoxal, which participates in neurotransmitter synthesis. Previous research has demonstrated that V9 can modulate sex hormone levels in patients with polycystic ovary syndrome via the gut–brain axis, a systemic regulatory capacity rarely observed in other probiotics. This further highlights the potential of V9 to exert multifaceted effects through metabolic–neural pathways [89]. Meanwhile, mounting clinical and preclinical evidence suggests that targeted microbiome interventions can improve mental health by modulating the gut–brain axis [90–92], including anxiety, depression, sleep quality, and overall quality-of-life scores. Multiple probiotic and synbiotic trials have reported improvements in emotional health and fatigue in patients with chronic inflammation or cancer [22, 93, 94], supporting the idea that microbiome-guided therapies can influence patients’ perceived health.

This study has notable strengths, including its double-blind, placebo-controlled design; longitudinal multi-omics integration (SGB-resolved metagenomics and untargeted LC-MS metabolomics); and use of validated clinical instruments (such as EORTC QLQ-C30). Nonetheless, limitations exist. The sample size (*n* = 104), while sufficient for primary endpoints, constrained power for subgroup analyses (e.g. gastric vs. esophageal cancer). Strict inclusion criteria, requiring completion of six chemotherapy cycles at the study centers, limited recruitment, as some patients transferred to local hospitals. Additionally, symptom assessment at Weeks 9 and 18 may have missed acute, cycle-dependent fluctuations in gastrointestinal toxicity. Importantly, although shotgun metagenomics was performed, our analyses primarily emphasized taxonomic (SGB-level) shifts. A more comprehensive functional characterization of bile acid, related metabolic pathways, such as bile salt hydrolase activity and secondary bile acid transformation, would strengthen mechanistic interpretation of the observed bile acid remodeling and its potential impact on mucosal physiology. Furthermore, this study did not include a direct comparison with standard chemotherapeutic adjuvants. In clinical practice, prophylactic supportive care, such as antiemetics and acid-suppressive agents, is ethically mandatory for patients with gastric and esophageal cancer undergoing chemotherapy. Therefore, our trial was designed to evaluate the additive value of V9 on top of standard supportive care, rather than to replace existing therapies. The absence of a head-to-head comparison limits our ability to assess the relative efficacy of V9 as a standalone adjuvant. Future trials should consider a three-arm design (standard supportive care vs. standard supportive care plus V9 vs. V9 monotherapy) combined with pharmacoeconomic evaluations to better define the positioning and clinical value of V9 in real-world oncology practice. Future studies integrating deeper functional annotation, pathway-level quantification, and host–microbe interaction analyses are warranted to clarify gene-level mechanisms linking microbiome modulation to chemotherapy tolerance. Larger, multicenter trials with higher-frequency symptom monitoring and expanded functional profiling will be essential to validate these findings and explore cancer type–specific responses.

The absence of serum-based multi-omics analysis represents a notable limitation of this study. Due to the study design, blood sample collection was specified as a nonmandatory option in the patient informed consent form, allowing patients to decide whether to participate based on personal willingness. Given the high prevalence of malnutrition and anemia among patients with advanced tumors, the majority declined blood collection, resulting in insufficient sample size and subsequent discontinuation of blood sampling during enrollment. This practical constraint precluded serum metabolomics and inflammatory cytokine profiling, thereby limiting our ability to directly verify whether gut-derived metabolites successfully entered the systemic circulation and mediated systemic effects. Furthermore, the lack of serum samples prevented direct measurement of inflammatory markers such as IL-6, TNF-α, and CRP. A central mechanism underlying gut–brain axis regulation involves changes in systemic inflammation resulting from improved intestinal barrier function. Although this study observed a reduction in opportunistic pathogens (e.g. *F. varium*) and an increase in barrier-protective metabolites (e.g. phosphatidylcholine), it could not confirm whether these local changes translated into reduced systemic inflammation. Future studies should prospectively incorporate paired fecal and serum sample collection, integrate multi-omics analyses with inflammatory marker monitoring, and systematically validate the hypothesized pathway of “microbiota remodeling—barrier repair—inflammation reduction—neurological improvement.”

Additionally, this study did not include functional validation using animal models. The causal relationships among key microbial taxa, their metabolites, and clinical phenotypes warrant further investigation in chemotherapy-induced animal models. Future research employing germ-free mice or antibiotic-treated models, combined with fecal microbiota transplantation and specific metabolite interventions, could elucidate the functional mechanisms of key bacteria and metabolites. Finally, as noted above, the absence of direct comparison with single-agent chemotherapy adjuvants limits assessment of the relative efficacy of V9.

## Conclusion

In summary, V9 improves quality of life in patients undergoing chemotherapy for advanced gastric or esophageal cancers through a coordinated mechanism: selective enrichment of beneficial commensals, suppression of pathobionts, and favorable reprogramming of the gut metabolome. These changes enhance mucosal integrity, reduce inflammation, and support neurological and metabolic resilience, ultimately alleviating both physical and psychological burdens of cancer therapy. This work provides a mechanistic foundation for microbiome-targeted supportive care and supports the integration of precision probiotics into oncology practice.

## Supplementary Material

Supplementary_Table_ycag127

## Data Availability

The original sequencing data supporting the conclusions of this study have been stored in the National Genomes Data Center, with the archive number CRA035632 (NGDC; https://ngdc.cncb.ac.cn/gsub/submit/gsa/subCRA058312/finishedOverview).
